# Anatomical distribution and gross pathology of wounds in necropsied farmed mink (*Neovison vison*) from June and October

**DOI:** 10.1186/s13028-016-0187-6

**Published:** 2016-01-25

**Authors:** Anna Jespersen, Jens Frederik Agger, Tove Clausen, Stine Bertelsen, Henrik Elvang Jensen, Anne Sofie Hammer

**Affiliations:** 1 Department of Veterinary Disease Biology, Faculty of Health and Medical Sciences, University of Copenhagen, Ridebanevej 3, 1870 Frederiksberg C, Denmark; 2 Kopenhagen Fur, Langagervej 60, 2600 Glostrup, Denmark; 3 Department of Large Animal Sciences, Faculty of Health and Medical Sciences, University of Copenhagen, Groennegaardsvej 8, 1870 Frederiksberg C, Denmark

**Keywords:** Mink, *Neovison vison*, Pathoanatomy, Season, Skin, Wound

## Abstract

**Background:**

Wounds are regarded as an indicator of reduced welfare in mink production; however, information on the occurrence and significance of wounds is sparse. To provide a basis for assessment and classification of wounds in farmed mink, the distribution pattern and characteristics of wounds in farmed mink in June and October, respectively, is described. A total of 791 and 660 mink from 6 to 12 Danish mink farms, respectively, were examined. The mink were either found dead or were euthanized due to injury or other disease. Mink included from June were kits in the pre-weaning and weaning period (1–2 months old). Mink included from October were juveniles in the late growth period (approximately 5–6 months old) or older. Macroscopic pathology and wound location was systematically recorded.

**Results:**

There was considerable variation in morphology as well as location of wounds between June and October. Wounds were primarily located on the front parts of the body and in the head in June (1–2 month old kits) and mainly on the rear parts of the body and on the tail in October (5–6 month old kits and older). Moreover, there were significantly more females than males with wounds for most wound types, and significant differences in occurrence of ear and tail base wounds between certain colour types.

**Conclusions:**

Wounds varied significantly from June to October with respect to morphology and anatomical location. Wounds in June were primarily located on the front parts of the body and in the head, while wounds in October were mainly present on the hind parts of the body and on the tail. The majority of the wounds were found in specific well defined skin areas and could therefore be grouped into categories according to anatomical location.

## Background

In Denmark and other mink producing countries, the impact of skin wounds and injuries on the welfare of mink has been in focus through resent years. Wounds are believed to be an indicator of reduced welfare in mink production due to pain and social stress [1]. Knowledge on the occurrence and significance of wounds in mink is sparse; however, Danish studies indicate, that around 10 % of mortality among mink kits is caused by bite wounds [2, 3]. The occurrence of wounds seem to increase in early and late growth season, respectively, and there may be differences in the causal mechanism between wounds in the early growth season and wounds occurring after weaning [3–5]. Due to the lack of knowledge about wounds in mink, management of wounds is carried out on a non-scientific background subjected to convenience and individual preferences. In other species, wounds are often characterized according to type, aetiology and degree of contamination [6]. Furthermore, for some species, specific assessment criteria have been defined for certain wound types or injuries, enhancing clinical handling of these lesions, e.g., shoulder wounds in sows [7] and hock lesions in cattle [8]. To provide a basis for wound assessment in mink, we have characterized wounds macroscopically according to anatomical distribution in dead farmed mink collected during two periods of the mink production cycle, i.e., the early and late growth season, i.e., June and October, respectively.

## Methods

The study was designed as a cross sectional study of dead mink collected continuously from 6 to 12 Danish mink farms over the months of June and October, respectively. The mink were either euthanized for welfare reasons or were found dead and stored in freezers until subsequent necropsy. Mink collected in June were all kits (1–2 months old) whereas mink from October included both juveniles and adults (5–6 months and older). The mink had been managed according to standard procedures following general legislation and guidelines for mink production. They were kept in standard cages with inserted kit wire mesh floors during the month of June, where the dam and kits were still together. From weaning until pelting in November the mink were kept in pairs or in groups of up to four mink in the same cage. A full necropsy was performed on all animals after thawing [9], specifically including registration of skin wounds. Registration of individual data including fur colour and sex was done for all animals. Wounds were examined macroscopically including registration of wound location, size (length × width measured in cases where the wound was not too irregular for correct wound area calculation), scab formation, granulation tissue formation, contraction, oedema, degree of infection, exudation/exudate type and condition of surrounding skin areas and wound edges including undermining of intact skin. Based on the location, wounds were categorized as: ear wounds, scalp wounds, neck wounds, shoulder wounds, thigh wounds, tail base wounds and tail tip wounds. Furthermore, there was a category of other wounds, i.e., less common wound locations that could not be placed in the before mentioned categories. A total of 791 mink from June and 1186 mink from October were examined. Of these, 526 pelted mink (without skin) from October were excluded from the dataset due to limited ability to identify wounds. The proportion of mink with different wound types in June and October and the distribution of wound types between sexes and colour types was calculated and presented graphically and in table form, respectively. If a mink had more than one wound, it might count for more wound types. Mink with injuries characterized as only post mortal due to the lack of tissue reaction (June: n = 216, October: n = 55) were solely included as part of the total number of mink for the calculation of proportions. Logistic regression was performed in SAS version 9.4 (SAS Institute, Cary, North Carolina, USA) using proc genmod for estimating significance of associations between wound type and sex and between wound type and colour using maximum likelihood parameter estimates for multiple comparisons of colour types. A *p* value of 0.05 was considered statistically significant.

## Results

**Fig. 1:**
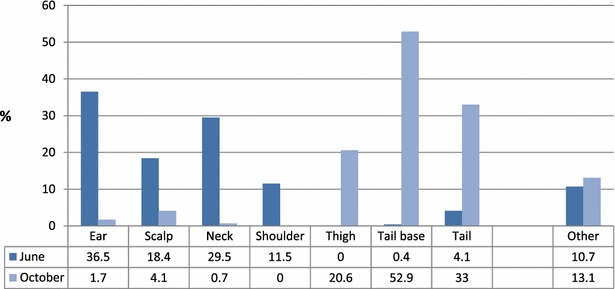
Distribution of wound types. Proportion of wound types (pathoanatomical) found in dead or euthanized mink with wounds on 6 farms (June, n = 244) and 12 farms (October, n = 291), respectively. Since mink may have more than one wound type, the percentages add up to more than 100 %

In total, one or more wounds were found in 244 (244/791 = 30.8 %) mink from June and 291 (291/660 = 44.1 %) mink from October. The seasonal distribution of lesion types (pathoanatomical) is illustrated in Fig. 1 which shows that most wounds in June were located on the front parts of the body and in the head, and that most wounds in October were located on the rear parts of the body and on the tail. Most wounds in June occurred in the second half of the month. In October the occurrence was more even over the entire month. Ear wounds (36.5 %) and neck wounds (29.1 %) were the most frequent location in June, whereas wounds at the base of the tail (37.3 %) and on the tail (23.2 %) were the most frequent location in October. Scalp wounds in June also include kits that were presumably killed by the dam by crushing of the skull (seven kits). Of animals with wounds, a total of 91.8 % and 94.8 % in June and October, respectively, had wounds categorized as ear, scalp, neck, shoulder, thigh, tail base or tail wounds. The distribution of the most common wound types (at least one wound per animal) in males and females, different colour types as well as mean wound size is given in Table 1. There were significantly more females with wounds than males for neck, shoulder, thigh, tail base and tail wounds, and significantly more ear wounds among males than females. There were significantly more black mink with ear wounds than both brown and white/light mink. White/light mink had also significantly fewer tail base wounds than both brown and blue/grey mink (Table 1).

**Table 1:** Distribution of sex and colour type and mean wound size for common mink wounds

Wound type (June)	Sex		Colour type					Mean wound size (cm^2^)
	♂ n = 320	♀ n = 325	Black n = 60	Brown n = 562	Blue/grey n = 18	White/light n = 102	Other n = 41	
Ear	46 (14.4 %)	20 (6.2 %)	12^a,b^ (20.0 %)	46^a^ (8.2 %)	2 (11.1 %)	3^b^ (2.9 %)	4 (9.8 %)	7.8 n = 53
	P = 0.001		P = 0.01					
Scalp	21 (6.6 %)	22 (6.8 %)	7 (11.7 %)	31 (5.5 %)	0 (0.0 %)	3 (2.9 %)	2 (4.9 %)	8.2 n = 34
	P = 0.916		P = 0.137					
Neck	15 (4.7 %)	52 (16.0 %)	4 (6.7 %)	55 (9.8 %)	2 (11.1 %)	7 (6.9 %)	4 (9.8 %)	8.7 n = 66
	P = 0.0001		P = 0.818					
Shoulder	3 (0.9 %)	25 (7.7 %)	1 (1.7 %)	20 (3.6 %)	0 (0.0 %)	3 (2.9 %)	4 (9.8 %)	7.8 n = 20
	P = 0.0001		P = 0.253					

Wound type (October)	Sex		Colour type					Mean wound size (cm^2^)
	♂ n = 283	♀ n = 355	Black n = 49	Brown n = 476	Blue/grey n = 25	White/light n = 81	Other n = 25	
Thigh	8 (2.8 %)	49 (13.8 %)	4 (8.2 %)	42 (8.8 %)	2 (8.0 %)	9 (11.1 %)	3 (12.0 %)	9.5 n = 80
	P = 0.0001		P = 0.946					
Tail base	19 (6.7 %)	131 (36.9 %)	8 (16.3 %)	118^a^ (24.8 %)	9^b^ (36.0 %)	10^a,b^ (12.3 %)	7 (28.0 %)	19.2 n = 141
	P = 0.0001		P = 0.031					
Tail	31 (11.0 %)	63 (17.7 %)	5 (10.2 %)	71 (14.9 %)	3 (12.0 %)	11 (13.6 %)	6 (24.0 %)	6.2 n = 22
	P = 0.015		P = 0.624					

Number and proportion of dead or euthanized mink presenting wounds on ear, scalp, neck and shoulder (June), or thigh, tail base and tail (October). The results are stratified according to sex and colour type. Mean wound size is given for each location. Due to occasional more wound types on the same mink and due to inability to determine the sex of all animals, the numbers may not be the same as the total proportions given in the text

^a,b^Estimates with the same letter are significantly different in multiple comparisons

**Fig. 2:**
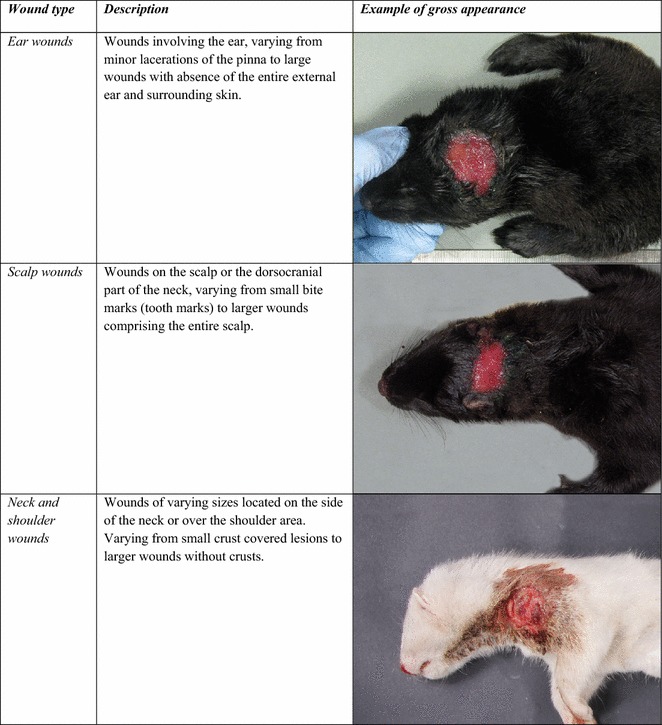
Pathoanatomical characteristics of wounds. Most common wounds seen in farmed mink in June

**Fig. 3:**
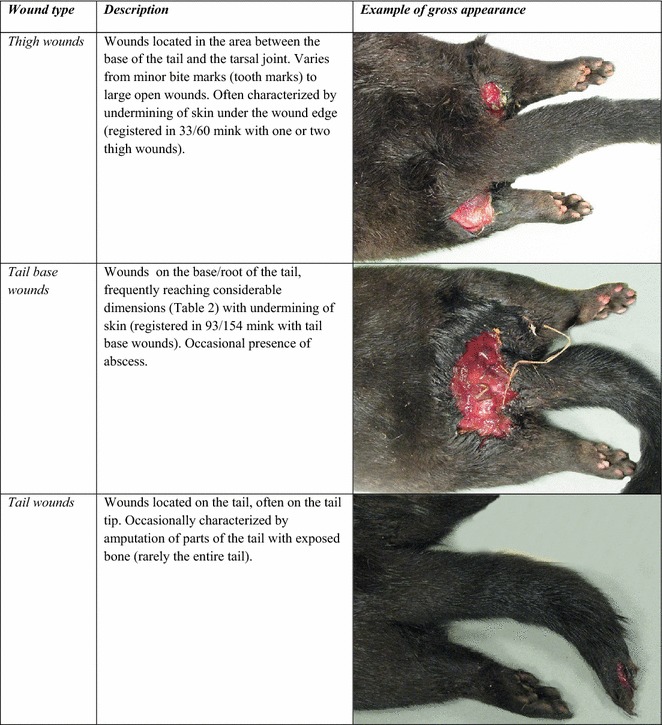
Pathoanatomical characteristics of wounds. Most common wounds seen in farmed mink in October

Pathologically, wounds varied according to anatomical location. In general, most wounds were not covered by a scab except for certain small wounds located on the neck or shoulders of mink kits which were covered by a thick semi-moist or greasy layer of exudate and debris. Signs of a reparative process like granulation tissue formation, wound contraction and epithelialization were seldom distinguishable, but oedema was usually present along the wound margins which were often irregular or frayed. The wounds were often contaminated and some showed signs of infection as defined by the presence of thick purulent exudate. Typical pathomorphological appearances of most frequent wound types are presented in Figs. 2 and 3. Due to the general lack of discernible reparative tissue, exact wound age could not be determined on macroscopic basis.

## Discussion

The development of a systematic basis for classification and clinical assessment of wounds in mink is necessary for the investigative efforts targeting control and prevention of wounds in this species. Though some mink with wounds may have died from other causes, the results are an indication of the proportion of mink that died or were euthanized due to skin lesions as opposed to other causes. Post mortal injuries may have erased signs of wounds occurring prior to death, contributing to an underestimation of frequencies. Moreover, pelted mink were excluded from the dataset which may have also led to a slight underestimation of the true proportion of dead wounded mink. The results are presented as frequencies and relative numbers and do not relate to the total number of mink on the farms.

Mink breed once each year and the kits are born late April or early May. The age of the majority of the mink present on the farm is therefore roughly the same throughout the year. The months June and October were selected for the study due to a report of increased frequency of wounds in these months compared to other periods in the mink production cycle [3]. This may be related to the age and developmental stage of the mink and management conditions related to the beginning and end of the growth season (i.e., weaning time and the period prior to pelting). As seen from Fig. 1, wounds were primarily located on the front parts of the body or in the head in June, while wounds in October were mostly located on the hind parts of the body and the tail. This likely represents the different behavioural mechanisms of the two age groups, leading to the formation of wounds during the two periods [4, 5]. Although some wound types may have overlapping morphological appearance, the distinct pathoanatomical distribution of wounds may reflect underlying risk or causal factors, similar to e.g., shoulder wounds in pigs [10] and breast/keel lesions in poultry [11]. Until further knowledge is at hand about the wound causal mechanisms in mink, it seems reasonable to maintain the proposed categories, and it is recommended that the classification of pathoanatomical location is included in future studies.

In June, the increasing occurrence of wounds through the end of the suckling period may be explained by the increased nutritional demands of the kits combined with a decline in the dam’s milk output and an increased competition for resources [12]. From early to mid-June, there will be increasing risk of bite wounds resulting from fights between siblings as part of this resource competition [4, 12]. In June, most wounds occurred from mid-month (6 weeks of age), which is in accordance with findings in other studies [12–14]. Dehydration or thirst may cause the well-known licking behaviour, where the kits lick or suck on the dam’s mouth [4, 15]. If this behaviour is directed towards the ears and other body parts of their siblings, it may lead to small lacerations or a moist dermatitis that may develop into wounds of varying size. The smaller wounds covered by a moist scab described in the neck and shoulder area may be such “lick-induced” lesions. A specific type of wound, rarely seen in the early growth period, is the characteristic crushing of the skull and tooth marks penetrating the scalp. They are inflicted to kits by the dam, presumably due to the dam’s frustration or stress from not being able to escape her kits demanding presence [16, 17].

Many of the wounds seen after weaning and until pelting, where the mink are housed in pairs (typically one male and one female) or in groups of up to four, are bite wounds resulting from aggression between cage mates [18]. The location of wounds on the mink’s hind parts may be interpreted as a flight/chase situation, where the bitten mink tries to escape the biting mink. The large size of wounds (Table 1) may be due to continuous biting since the submissive mink cannot escape. The submissive mink will likely be the smaller of the pair/group, which is supported by the findings of a larger proportion of wounds in females than in males (Table 1). Previous studies have demonstrated a similar ratio of lesions in females compared to males [3, 12, 17]. Similarly, the dominance of ear wounds in males has been demonstrated previously [12]. For ear wounds white/light and brown mink had significantly fewer wounds than black mink. For tail base wounds, white/light mink had significantly fewer wounds than both brown and blue/grey mink (Table 1). The reason for this is unknown, but may reflect behavioural and temperamental differences between the colour types [19]. Apart from the specific location, wounds found during October were often associated with undermining of skin which may result from ruptured abscesses, i.e., similar to what is often seen in cats [20]. This may also explain the large mean size of wounds, especially on the base of the tail. In areas where skin is not firmly adhered to the underlying tissues, initially the tension and resilience of the skin causes the wounds to widen once inflicted.

Accurate assessment of wound age was not possible based on gross pathology and would require histological examination. Although wound healing in mink is expected to include the same phases as in other mammals, no detailed studies of mink wound pathology have been published so far. It was considered out of the scope of this report to present histopathological results. The general failure to identify signs of reparation may be explained by quick discovery and euthanasia of wounded mink by farm personnel or that mink often die from their wounds in the acute stage.

In conclusion, we found a significant seasonal variation in location of wounds in farmed mink. Wounds in June were mainly situated on the front parts of the body and in the head, while wounds in October were mainly present on the hind parts of the body and on the tail. There were significant differences in occurrence between males and females, and for some wound types differences between certain colour types. The results may provide a basis for further studies of the cause and mechanisms behind different wound types as well as for developing guidelines for wound assessment in mink. Furthermore, the pathoanatomical wound distribution may define which wound types to focus on in future studies of prevention and management of wounds in farmed mink.
